# Difference in eye movements during gait analysis between professionals and trainees

**DOI:** 10.1371/journal.pone.0232246

**Published:** 2020-04-30

**Authors:** Kazuhiro Hayashi, Shuichi Aono, Mitsuhiro Fujiwara, Yukiko Shiro, Takahiro Ushida

**Affiliations:** 1 Multidisciplinary Pain Center, Aichi Medical University, Nagakute, Japan; 2 Department of Rehabilitation, Aichi Medical University Hospital, Nagakute, Japan; 3 Department of Pain Data Management, Aichi Medical University, Nagakute, Japan; 4 Department of Rehabilitation, Kamiiida Rehabilitation Hospital, Nagoya, Japan; 5 Department of Physical Therapy, Faculty of Rehabilitation Sciences, Nagoya Gakuin University, Nagoya, Japan; The Education University of Hong Kong, HONG KONG

## Abstract

**Introduction:**

Observational gait analysis is a widely used skill in physical therapy. Meanwhile, the skill has not been investigated using objective assessments. The present study investigated the differences in eye movement between professionals and trainees, while observing gait analysis.

**Methods:**

The participants included in this study were 26 professional physical therapists and 26 physical therapist trainees. The participants, wearing eye tracker systems, were asked to describe gait abnormalities of a patient as much as possible. The eye movement parameters of interest were fixation count, average fixation duration, and total fixation duration.

**Results:**

The number of gait abnormalities described was significantly higher in professionals than in trainees, overall and in limbs of the patient. The fixation count was significantly higher in professionals when compared to trainees. Additionally, the average fixation duration and total fixation duration were significantly shorter in professionals. Conversely, in trunks, the number of gait abnormalities and eye movements showed no significant differences between groups.

**Conclusions:**

Professionals require shorter fixation durations on areas of interest than trainees, while describing a higher number of gait abnormalities.

## Introduction

Physical therapy provides to improve maximum movement, functional ability, and quality of life for patients. Gait analysis is used to diagnose gait disorders that are caused by musculoskeletal and neurological system pathologies and determine correct and effective physical therapy programs. [1–4] Computerized three-dimensional gait analysis technology is not commonly available in clinical practice, its application is complex and time demanding, and clinicians are often unfamiliar with its results and terminology. [1–4] Observational gait analysis is the most widely used method of gait analysis in clinical settings because it is a fast, simple and inexpensive method. [1–4] It should be noted that observational gait analysis is a learned skill because it requires a wide variety of movements of different parts of the body at the same time. Physical therapist trainees desire to improve their observational gait analysis skills as much as possible.

Eye tracking technology shows an objective assessment of skills, better than the subjective assessments (e.g., verbal reports, or text books for students) that are currently available. [5] Eye tracking is an accurate and reliable way of measuring an eye movements and reflects their attention allocation. [6, 7] For example, the eye remains relatively stable when the attention is on an object. The eyes move rapidly from one area to another along with change in attention. A fixation means that the eye pauses on a specific area of the visual field (relatively stable eye movement), whereas saccades are rapid eye movements from one fixation to another. [6] Eye tracking technology could reveal how professionals are able to solve complex tasks after glancing very briefly, and therefore it provides insight in guiding learners. [5] Numerous studies with eye tracking technologies compare the differences in eye movement parameters between professionals and novices. [5] The differences have been mostly investigated in sports, reading, and psychology. [5] Following them, some studies have investigated the usefulness of eye tracking technology in clinical practices. [5, 7–9] Eye movement is shown to be associated with medical diagnostic accuracy and efficiency. [10,11] As recent technology advances, several eye movement studies have been conducted in static and dynamic medical settings; specifically, studies have been conducted in surgery, [12–16] dynamic presentation of the radiographic images, [17,18] and neurology. [19] According to results of these studies, [12–19] medical and related professional physicians often require a fewer number of fixations and less time spent on areas of interest, while having a higher rate of accuracy than trainees. [5, 7–9] Their findings are task-specific for a learned skill. [20] These data suggest that professionals are able to quickly identify suspicious regions at low magnification, [5, 7–9] although features were typically shown in a small number of participants. [5] Meanwhile, there are few studies investigated with eye movements to patient behavior, among medical expertise. Patient behavior and motion are quite variable. The eye movement while performing an observational gait analysis has not been investigated yet. The observational gait analysis has been investigated using a self-administered questionnaire for observers, [1–4] instead of an objective assessment.

Thus, the present study investigated the differences in the eye movement between professional physical therapists and physical therapist trainees, through observational gait analysis. It is hypothesized that professional physical therapists require a fewer number of fixations on areas of interest than physical therapist trainees. The differences in eye movement between professionals and trainees are hypothesized to be more prominent in body regions with difficult gait abnormalities.

## Materials and methods

### Experimental design

An observational design was used to investigate the differences in eye movement during observational gait analysis between professionals and trainees. First, gait motion of a patient was recorded in a video clip. Subsequently, the observers (i.e. professional physical therapists and physical therapist trainees) who were wearing eye tracker systems performed gait analysis of the patient using the video clip.

This study was approved by the Ethics Committee of Nagoya Gakuin University and Kamiiida Rehabilitation Hospital. All experiments were carried out from June 2018 to November 2018.

### Observer groups

We recruited 26 professional physical therapists in two medical institutions and 26 physical therapist trainees in one university to participate in the present study by means of posting flyers on a notice board. All participants provided written informed consent prior to participation in this study.

The 26 professional physical therapists were full-time physical therapists who had extensive experience interpreting gait performances, ranging from three to seven years. The professional physical therapists evaluated gait analyses every day during physical therapy and had already passed the program for new employees.

The 26 physical therapist trainees were final year students of an undergraduate program in physical therapy. The trainees had passed the curriculum and clinical trainings for patient assessments, including gait analysis, in their colleges, and the clinical practicum for physical therapy. However, the trainees had not yet taken the national exam for physical therapy and did not have clinical experience in physical therapy.

The sample size was calculated using G*Power software (v3.1.9.2; Franz Faul, Universität Kiel, Kiel, Germany). Based on an effect size of 0.8 for the measurement of eye tracking, [14–17] the minimum number of subjects required for each group was estimated to be 26, resulting in an α–level of .05 and a power (1−β) of .80.

### Video clip record of gait motion of a patient

A video recording of gait motion for one patient was tracked in a medical institution. The patient required regular rehabilitation for abnormal gait motion with flaccid hemiplegia. The flaccid hemiplegia was manifested by the softness and weakness of the affected muscles. This patient had independent gait with walking aids, even with severe impairment (Brunnstrom motor recovery stage 5 for upper extremity function, stage 5 for lower extremity function, [21] and mild hypoesthesia).

The patient was asked to walk along a 20-meter straight-line path, at a comfortable self-selected gait speed (.87 m/sec, for approximately 23 sec). The patient was recorded from anterior by a camera. The patient’s face was pixelated for confidentiality.

### Videotaped observational gait assessment

The videotaped observational gait assessment was performed in a laboratory. [1–4] The present study used free describing methods of gait abnormalities instead of gait assessment tools. The observers watched the video clip for gait assessment. After they had finished watching the video clip, they described gait abnormalities of the patient as many as possible.

Observers were seated in front of a screen while wearing a head-mounted eye tracker. Observers blindly performed gait analysis for the video clip, without having access to other medical information. Gait descriptions for the patient were checked for accuracy compared to a model answer by two professional therapists using slow motion on the video clip. The number of correct descriptions of gait abnormalities was calculated and these abnormalities were classified (e.g., limb, trunk, walking aids, and center of gravity), because the descriptions included contrast, timing, and causal link among body regions.

### Measurement of eye movement

Eye movement data were acquired using eye tracking glasses (Tobii Pro Glasses 2, Tobii Technology, Danderyd, Sweden), and were calibrated to each individual participant (Tobii Pro Glasses Controller, Tobii Technology, Danderyd, Sweden). [22,23] The sampling frequency in the eye tracker was a 50 Hz. Eye tracking glasses used near-infrared illumination to create reflection patterns on the cornea and pupil. Two image sensors were used to capture images of the eyes and the reflection patterns. They were used to estimate the position of the eye in space, as well as the point of gaze. A nine point calibration was done prior to each measurement to ensure good quality of the eye gaze recordings. Observers were instructed to watch the screen, therefore they were not allowed to look elsewhere during the recording.

The eye movement data were analyzed using analysis software (Tobii Glasses Analysis software, Tobii Technology, Danderyd, Sweden). [22,23] The areas of interest accurately specified areas on the patient video (e.g., head and neck, non-paralyzed lower side limb, non-paralyzed upper side limb, paralyzed lower side limb, paralyzed upper side limb, lower trunk, upper trunk, or walking aids) were defined. The parameters obtained from the eye tracking analysis were fixation count, average fixation duration, and total fixation duration. A fixation was identified as when the mean horizontal and vertical eye position co-ordinates sustained eye movement at a location within 30°/s of the visual angle. The count was the number of fixations within each area of interest. The average fixation duration was the average of each fixation duration within each area on interest. The total fixation duration was the sum of all fixation durations for each area of interest. All measurements ensured good quality of the eye gaze recordings without removing the data.

### Statistical analysis

Normality for each measurement was evaluated using the Shapiro-Wilk test for continuous variables. The outcome variables were not normally distributed; all continuous data are expressed as medians and interquartile ranges (IQR).

The statistical differences between the groups were analyzed using the Mann-Whitney U test. All data were statistically analyzed using the SPSS 24.0J program. An effect size (r) was calculated to determine the magnitude of difference between groups. It was constrained to lie between 0 (no effect) and 1 (a perfect effect). An effect size (r) of ≥0 and <0.1 was classified as no effect, ≥0.1 and <0.3 as a small effect, ≥0.3 and <0.5 as a moderate effect, and ≥0.5 as a large effect. A P-value of < .05 was considered statistically significant.

## Results

### Videotaped observational gait assessment

**Fig 1:**
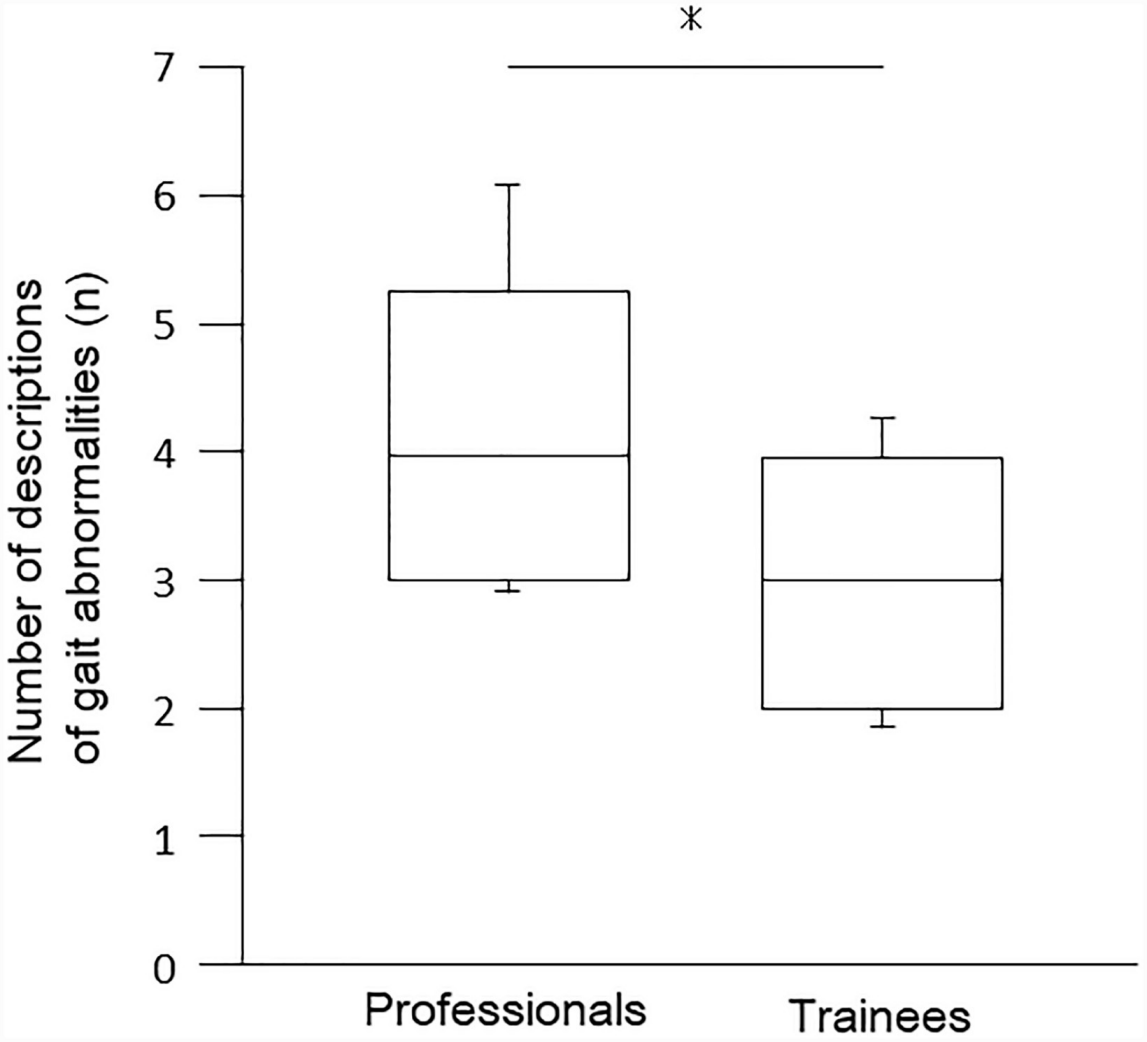
The number of descriptions of gait abnormalities. Values are expressed as medians and interquartile ranges (IQR) with standard deviation error bars. Asterisk shows statistical significance, *: p < .05 professionals vs. trainees.

Fig 1 and Table 1 show the number of correct descriptions of gait abnormalities; there were no incorrect descriptions in either group. The number of descriptions of gait abnormalities was significantly higher in professionals (median, 4; IQR, 3–5) than in trainees (median, 3; IQR, 2–4) (moderate effect size).

**Table 1:** The number of descriptions of gait abnormalities.

	Professionals (n = 26)	Trainees (n = 26)	P-value	Effect size (r)
Overall	4 [3–5]	3 [2–4]	.001*	.47
Limb	3 [3–4]	3 [2–3]	.005*	.41
Trunk	1 [0–1]	0 [0–1]	.369	.15
Walking aids	0 [0–0]	0 [0–0]	---	---
Center of gravity	0 [0–1]	0 [0–0]	.313	.14

The number of descriptions of gait abnormalities are shown as median [IQR].

Asterisk shows statistical significance,

*: p < .05.

The number of descriptions of gait abnormalities was significantly higher in professionals than in trainees.

Particularly, the number of descriptions was significantly higher in limbs (median, 3; IQR, 3–4) in professionals (e.g., knee hyperextension during the stance phase, and decrease in knee flexion during the swing phase; in addition to drop foot) than in trainees (e.g., drop foot) (limbs; median, 3; IQR, 2–3) (moderate effect size). There were no significant differences in trunk and center of gravity between professionals and trainees (e.g., lateral trunk flexion).

### Fixation parameters from eye tracking

**Fig 2:**
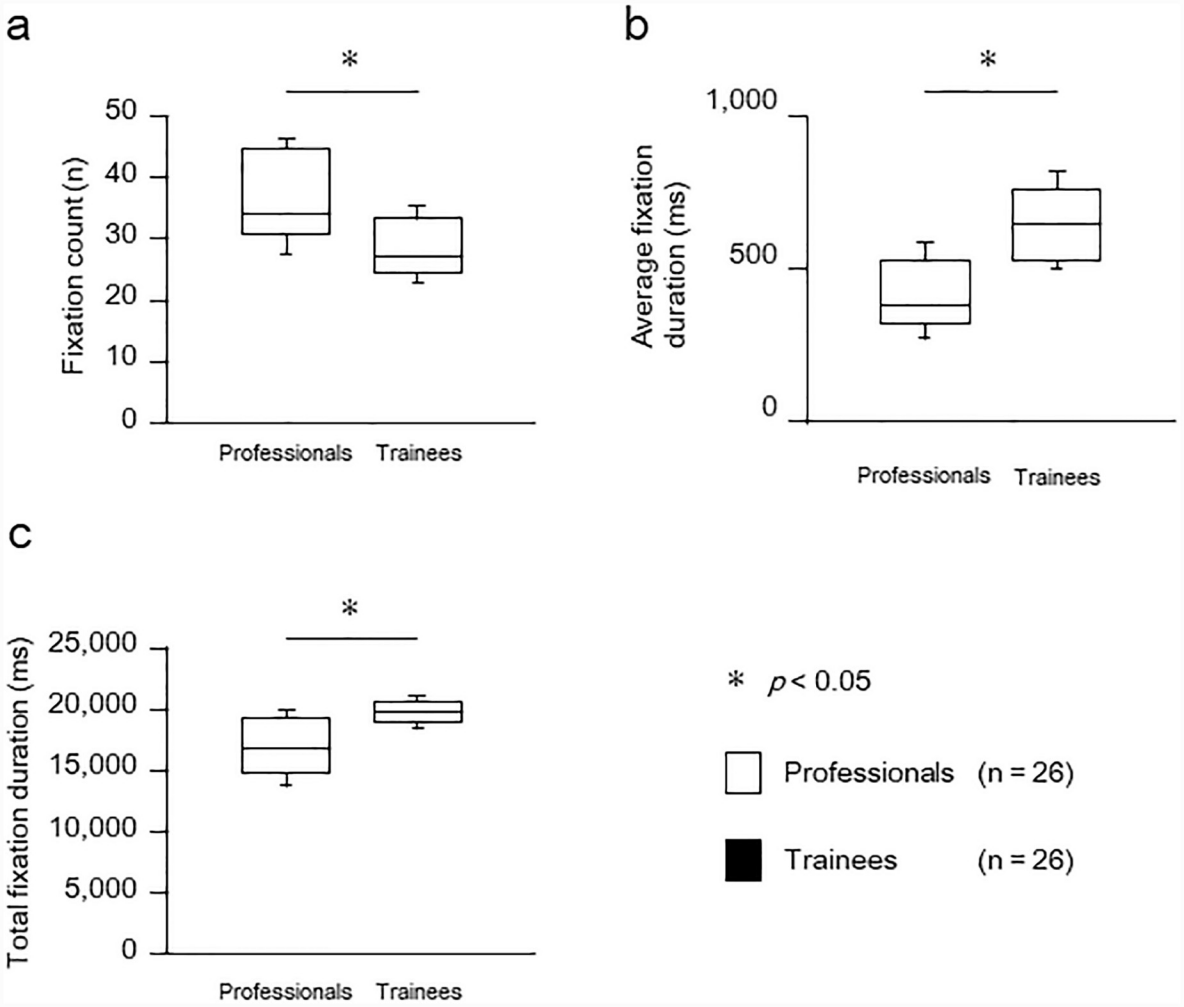
Fixation parameters from eye tracking, overall of the patient. (a) Fixation count. (b) Average fixation duration. (c) Total fixation duration. Values are expressed as medians and interquartile ranges (IQR) with standard deviation error bars. Asterisk shows statistical significance, *: p < .05 professionals vs. trainees.

Fig 2 and Table 2 show fixation parameters from eye tracking analysis. The fixation count was significantly higher in professionals than in trainees (professionals; median, 35; IQR, 31–45) (trainees; median, 28; IQR, 25–34) (moderate effect size). The average fixation duration and the total fixation duration were significantly shorter in professionals (average fixation duration (ms); median, 387; IQR, 331–533) (total fixation duration (ms); median, 16,936; IQR, 15,027–19,380) than in trainees (average fixation duration; median, 652; IQR, 530–767) (total fixation duration; median, 19,835; IQR, 19,020–20,805) (large effect size in both measurements).

**Table 2:** Fixation parameters from eye tracking.

	Professionals (n = 26)	Trainees (n = 26)	P-value	Effect size (r)
*Fixation Count*, *n*				
Overall	35 [31–45]	28 [25–34]	.002*	.46
Head and neck	0 [0–0]	0 [0–0]	.080	.32
Non-paralyzed lower side limb	13 [9–16]	9 [6–11]	.007*	.42
Non-paralyzed upper side limb	0 [0–2]	1 [0–2]	.390	.05
Paralyzed lower side limb	12 [10–19]	9 [6–11]	.004*	.40
Paralyzed upper side limb	1 [0–3]	0 [0–1]	.042*	.14
Lower trunk	3 [2–5]	4 [2–6]	.547	.15
Upper trunk	1 [0–5]	3 [2–5]	.123	.19
Walking aids	1 [0–2]	0 [0–0]	.002*	.48
*Average Fixation Duration*, *ms*				
Overall	387 [331–533]	652 [530–767]	< .001*	.59
Head and neck	0 [0–0]	0 [0–0]	.072	.23
Non-paralyzed lower side limb	410 [326–647]	728 [553–926]	< .001*	.52
Non-paralyzed upper side limb	0 [0–265]	247 [0–462]	.094	.28
Paralyzed lower side limb	544 [401–682]	657 [562–873]	.005*	.43
Paralyzed upper side limb	150 [0–245]	0 [0–317]	.286	.09
Lower trunk	354 [253–511]	492 [364–868]	.019*	.36
Upper trunk	193 [0–440]	535 [290–894]	.002*	.32
Walking aids	125 [0–537]	0 [0–0]	.004*	.40
*Total Fixation duration*, *ms*				
Overall	16,936 [15,027–19,380]	19,835 [19,020–20,805]	< .001*	.61
Head and neck	0 [0–0]	0 [0–0]	.072	.29
Non-paralyzed lower side limb	4,739 [3,334–7,208]	6,448 [4,219–8,253]	.120	.18
Non-paralyzed upper side limb	0 [0–375]	380 [0–1,265]	.195	.07
Paralyzed lower side limb	6,888 [4,979–9,937]	6,858 [3,659–9,383]	.728	.05
Paralyzed upper side limb	240 [0–745]	0 [0–440]	.127	.02
Lower trunk	1,310 [855–2,469]	2,459 [1,515–3,149]	.027*	.39
Upper trunk	490 [0–2,174]	1,840 [780–3,209]	.011*	.37
Walking aids	210 [0–1,380]	0 [0–0]	.002*	.51

Fixation parameters from eye tracking are shown as median [IQR].

Asterisk shows statistical significance,

*: p < .05.

The fixation count was significantly higher in professionals than in trainees. The average fixation duration and total fixation duration were significantly shorter in professionals than in trainees

Similarly, the limbs and trunks tended to have a shorter average fixation duration in professionals than in trainees, although fixation count in the trunks was consistent between groups. Conversely, the walking aids were almost not seen in trainees, and tended to be associated with fewer fixation counts and shorter fixation durations than in professionals.

## Discussion

The present study showed that professionals require shorter fixation durations on areas of interest, while performing a higher number of gait abnormality descriptions than trainees, overall and in limbs of the patient. Conversely, in trunks, the number of gait abnormality descriptions and fixation counts showed no significant differences between groups. The present study is the first to show the differences in eye movements during gait analysis between professionals and trainees.

Professionals can quickly identify suspicious regions at low magnification and then spontaneously identify diagnostically relevant features, resulting in shorter fixation duration and less time spent on areas of interest. [5, 7–9] Notably, expertise studies using eye tracking methodology in medical settings had small sample sizes, which averaged eight patients. [5] A small number of participants in each group can produce sampling error. Similarly, the present study showed shorter fixation durations on areas of interest in professionals than trainees, while performing an observational gait analysis, with a larger, more appropriate sample size. In addition, the fixation count tended to be higher in professionals than trainees. This result suggests that professionals frequently pay attention to different areas, while spending less time in each. The fixation is suggested to be at least two types of ambient and focal. [24,25] An ambient attention, which is recognized by relatively short fixations and long saccades, is indicator of the pre attentive process of exploring the spatial organization of the visual scene. A focal attention, which is recognized by relatively long fixations and short saccades, is associated with focused attention on the object. The types of fixation during gait analysis have not been investigated in observational gait analysis between professionals and trainees.

The differences in eye movement between professionals and trainees are more prominent in cases with inherently higher diagnostic difficulty. [26] Similarly, in overall areas of interest and limbs of the present study, professionals described more gait abnormalities with shorter fixation durations than trainees. Meanwhile, in trunks, fixation count showed no significant differences between groups. The number of descriptions of gait abnormalities in trunks was less and equivalent in both groups. Professionals may estimate the trunks are not crucial or difficult for gait assessment of the patient, while trainees could not estimate them. The brain responses in observational gait analysis have not been investigated using eye fixation-related potential method. [27]

The walking aids were almost not seen in trainees, although the professionals showed minimal fixation durations. As a result, some professionals (15%, n = 4/26) described gait abnormalities in walking aids; however, no trainees described it. The other professionals and trainees would need to expand attention allocation.

Eye tracking technology provides information about an individual’s attention allocation over time, based on mechanisms used to follow eye behavior. [6, 7] The eye movement of professionals focuses on closer key target areas, whereas that of novice individuals often wander from key areas. [12, 17, 19] Professionals achieve a significantly higher level of overlap of eye movement than novices. [12] The present study also showed the fixation count was significantly higher in professionals than in trainees. There are three theories that may explain eye movement differences; that is, the theory of long-term working memory, the information-reduction hypothesis, and the holistic model of image perception. [5] These theories provide complementary accounts of some of the mechanisms underlying the reproducibility of professional task superiority. [5]

The aging society is in rapid progress all over the world, with Japan in the lead. [28] The number of new physical therapists is almost eight times as compared to two decades ago in Japan, along with a prolongation of life expectancy. [29] There is a need to improve the efficiency of training and use new educational techniques. The differences in eye movements during gait analysis between professionals and trainees could encourage the trainees to understand the skill of observational gait analysis. The instructional video shows how an attentional focus of the professionals based on eye tracking technology is useful in medical education; [30] however, it has not been reported in gait analysis. Additionally, the eye movements have a possibility to assess the skill of observational gait analysis in trainees.

There are several limitations in the present study. First, the video clip was recorded only from anterior of one flaccid hemiplegia patient. Some gait descriptions could be lacking, because the video only shows the anterior view and not posterior or lateral views. Second, we recruited limited professional physical therapists and physical therapist trainees. The gait analysis skills might differ from experience years or amount of education in school and clinical settings. Third, observational gait analysis skill is only a part of skills obtained by physical therapists. Professional physical therapists use other skills to get information from some physical movements, medical information, medical interviews, and others. Various skills are required to improve maximum movement, functional ability, and quality of life for patients. Finally, eye movement during videotaped observational gait assessment was measured using a head-mounted eye tracker. A remote eye tracker is suitable to measure the eye movement while watching a video clip.

## Conclusions

The present study showed that professional physical therapists require shorter fixation durations on areas of interest, while providing a higher number of descriptions of gait abnormalities than physical therapist trainees.

## Supporting information

S1 Data (XLSX)

## References

[1] Ferrarin M, Rabuffetti M, Bacchini M, et al Does gait analysis change clinical decision-making in poststroke patients? Results from a pragmatic prospective observational study. Eur J Phys Rehabil Med. 2015; 51: 171–184. 25184798

[2] Gor-García-Fogeda MD, Cano de la Cuerda R, Carratalá Tejada M, et al Observational Gait Assessments in People With Neurological Disorders: A Systematic Review. Arch Phys Med Rehabil. 2016; 97: 131–140. 10.1016/j.apmr.2015.07.018 26254954

[3] Ferrarello F, Bianchi VA, Baccini M, et al Tools for observational gait analysis in patients with stroke: a systematic review. Phys Ther. 2013; 93: 1673–1685. 10.2522/ptj.20120344 23813091

[4] Brunnekreef JJ, van Uden CJ, van Moorsel S, Kooloos JG. Reliability of videotaped observational gait analysis in patients with orthopedic impairments. BMC Musculoskelet Disord. 2005; 17; 6: 17. 10.1186/1471-2474-6-17 PMC555760 15774012

[5] Gegenfurtner A, Lehtinen E, Säljö R. Expertise differences in the comprehension of visualizations: A meta-analysis of eye-tracking research in professional domains. Educ Psychol Rev. 2011; 23: 523–552.

[6] Holmqvist K, Andrà C, Lindström P, et al A method for quantifying focused versus overview behavior in AOI sequences. Behav Res Methods. 2011; 43: 987–998. 10.3758/s13428-011-0104-x 21557008

[7] Ashraf H, Sodergren MH, Merali N, Mylonas G, Singh H, Darzi A. Eye-tracking technology in medical education: A systematic review. Med Teach. 2018; 40: 62–69. 10.1080/0142159X.2017.1391373 29172823

[8] Harezlak K, Kasprowski P. Application of eye tracking in medicine: A survey, research issues and challenges. Comput Med Imaging Graph. 2018; 65: 176–190. 10.1016/j.compmedimag.2017.04.006 28606763

[9] Al-Moteri MO, Symmons M, Plummer V, Cooper S. Eye tracking to investigate cue processing in medical decision-making: A scoping review. Comput Human Behav. 2017; 66: 52–66.

[10] Voisin S, Pinto F, Morin-Ducote G, Hudson KB, Tourassi GD. Predicting diagnostic error in radiology via eye-tracking and image analytics: preliminary investigation in mammography. Med Phys. 2013; 40: 101906 10.1118/1.4820536 24089908

[11] Brunyé TT, Mercan E, Weaver DL, Elmore JG. Accuracy is in the eyes of the pathologist: The visual interpretive process and diagnostic accuracy with digital whole slide images. J Biomed Inform. 2017; 66: 171–179. 10.1016/j.jbi.2017.01.004 28087402 PMC5316368

[12] Khan RS, Tien G, Atkins MS, Zheng B, Panton ON, Meneghetti AT. Analysis of eye gaze: do novice surgeons look at the same location as expert surgeons during a laparoscopic operation? Surg Endosc. 2012; 26: 3536–3540. 10.1007/s00464-012-2400-7 22733194

[13] Ahmidi N, Ishii M, Fichtinger G, Gallia GL, Hager GD. An objective and automated method for assessing surgical skill in endoscopic sinus surgery using eye-tracking and tool-motion data. Int Forum Allergy Rhinol. 2012; 2: 507–515. 10.1002/alr.21053 22696449

[14] Harvey A, Vickers JN, Snelgrove R, Scott MF, Morrison S. Expert surgeon's quiet eye and slowing down: expertise differences in performance and quiet eye duration during identification and dissection of the recurrent laryngeal nerve. Am J Surg. 2014; 207: 187–193. 10.1016/j.amjsurg.2013.07.033 24476801

[15] Kocak E, Ober J, Berne N, Melvin WS. Eye movement parameters correlate with level of experience in video-assisted surgery: Objective testing of three tasks. J Laparoendosc Adv Surg Tech A. 2005; 15: 575–580. 10.1089/lap.2005.15.575 16366861

[16] Wilson M, McGrath J, Vine S, Brewer J, Defriend D, Masters R. Psychomotor control in a virtual laparoscopic surgery training environment: gaze control parameters differentiate novices from experts. Surg Endosc. 2010; 24: 2458–2464. 10.1007/s00464-010-0986-1 20333405 PMC2945464

[17] Bertram R, Helle L, Kaakinen JK, Svedström E. The effect of expertise on eye movement behaviour in medical image perception. PLoS One. 2013; 13; 8:e66169. 10.1371/journal.pone.0066169 PMC3681771 23785481

[18] Mallett S, Phillips P, Fanshawe TR, et al Tracking eye gaze during interpretation of endoluminal three-dimensional CT colonography: visual perception of experienced and inexperienced readers. Radiology. 2014; 273: 783–792. 10.1148/radiol.14132896 25028782

[19] Balslev T, Jarodzka H, Holmqvist K, et al Visual expertise in paediatric neurology. Eur J Paediatr Neurol 2012; 16: 161–166. 10.1016/j.ejpn.2011.07.004 21862371

[20] Kelly B, Rainford LA, McEntee MF, Kavanagh EC. Influence of radiology expertise on the perception of nonmedical images. J Med Imaging (Bellingham) 2018; 5: 031402. 29250569 10.1117/1.JMI.5.3.031402 PMC5724551

[21] Brunnstrom S. Motor testing procedures in hemiplegia: based on sequential recovery stages. Phys Ther 1966; 46: 357–375. 10.1093/ptj/46.4.357 5907254

[22] Tien T, Pucher PH, Sodergren MH, Sriskandarajah K, Yang GZ, Darzi A. Eye t racking for skills assessment and training: a systematic review. J Surg Res. 2014; 191: 169–178 10.1016/j.jss.2014.04.032 24881471

[23] Tobii technology. User Manual (2003), http://www.tobii.se Accessed 25 Mar 2020

[24] Krejtz K, Coltekin A, Duchowski AT, Anna N. Using coefficient K to distinguish ambient/focal visual attention during cartographic tasks. J Eye Mov Res. 2017; 10: 1–13. 10.16910/jemr.10.2.3 PMC7141058 33828650

[25] Follet B, Le Meur O, Baccino T. New insights into ambient and focal visual fixations using an automatic classification algorithm. Iperception. 2011; 2: 592–610. 10.1068/i0414 23145248 PMC3485802

[26] Brunyé TT, Carney PA, Allison KH, Shapiro LG, Weaver DL, Elmore JG. Eye movements as an index of pathologist visual expertise: a pilot study. PLoS One. 2014; 1; 9: e103447. 10.1371/journal.pone.0103447 PMC4118873 25084012

[27] Fudali-Czyż A, Francuz P, Augustynowicz P. The Effect of Art Expertise on Eye Fixation-Related Potentials During Aesthetic Judgment Task in Focal and Ambient Modes. Front Psychol. 2018; 9:1972 10.3389/fpsyg.2018.01972 30459676 PMC6232682

[28] World Population Prospects. https://population.un.org/wpp/ Accessed 25 Mar 2020

[29] About Japanese Physical Therapy Association. http://www.japanpt.or.jp/english/international/for-foreigner/english/ Accessed 25 Mar 2020

[30] Jarodzka H, Balslev T, Holmqvist K, et al Conveying clinical reasoning based on visual observation via eye-movement modelling examples. Instructional Science 2012; 40:813–827.

